# Multicentric Asynchronous Endocrine Mucin‐Producing Sweat Gland Carcinoma and Mucinous Carcinoma of the Skin

**DOI:** 10.1111/cup.70122

**Published:** 2026-04-29

**Authors:** Ikuko Hirai, Joseph F. Sobanko, Tara C. Mitchell, Jacquelyn Roth, Rosalie Elenitsas, Emily Y. Chu

**Affiliations:** ^1^ Department of Dermatology Hospital of the University of Pennsylvania Philadelphia Pennsylvania USA; ^2^ Department of Dermatology Keio University of School of Medicine Tokyo Japan; ^3^ Division of Hematology/Oncology, Department of Medicine Hospital of the University of Pennsylvania Philadelphia Pennsylvania USA; ^4^ Department of Pathology and Laboratory Medicine Hospital of the University of Pennsylvania Philadelphia Pennsylvania USA

**Keywords:** endocrine mucin‐producing sweat gland carcinoma, mucinous carcinoma of the skin, multicentric, sweat gland tumor

## Abstract

Endocrine mucin‐producing sweat gland carcinoma (EMPSGC) is an adnexal neoplasm which typically occurs on periorbital skin and demonstrates overlapping histopathologic features with primary mucinous carcinoma of the skin (MCS). Herein, we report a patient who developed five distinct lesions of EMPSGC and MCS over an eight‐year period, some of which occurred on periorbital sites and others which occurred on the lower face. We further sought to better characterize these tumors on a molecular basis; targeted next generation sequencing analysis of two discrete tumors revealed identical missense variants within the *ATRX* gene. Our report expands on the rare prior reports of multicentric, asynchronous EMPSGC and MCS, and also provides a basis for additional genetic classification of these neoplasms.

## 1 Introduction

Endocrine mucin‐producing sweat gland carcinoma (EMPSGC) was first described by Flieder et al. in 1997 as an adnexal neoplasm with a predilection for the periorbital skin and morphologically analogous to solid papillary carcinoma of the breast (SPC) [1]. Both are low‐grade solid/cystic adenocarcinomas with mucin production, express neuroendocrine markers in various degrees and demonstrate histopathologic overlap with invasive mucinous carcinoma.

Several recent reports have highlighted an uncommon presentation of these tumors: multicentricity [2, 3, 4, 5, 6, 7]. The mechanism underlying multicentric and asynchronous EMPSGCs is unknown. A next‐generation sequencing (NGS) study (MSK‐IMPACT, 368 genes) identified somatic mutations in DNA repair and tumor suppressor pathways such as BRD4, PPP4R2, and TP53 in EMPSGC, which may be implicated in the multistep molecular pathogenesis of EMPSGC [8]. However, to our knowledge, no studies have focused on the clonality of multiple EMPSGC/mucinous carcinoma of the skin (MCS) lesions in an individual via the homogeneity and heterogeneity of genetic profiles.

Herein, we report a patient with an 8‐year history of five lesions of EMPSGC and MCS widely distributed beyond the periorbital region on the face, along with the NGS results from several lesions.

## 2 Case Report

**FIGURE 1:**
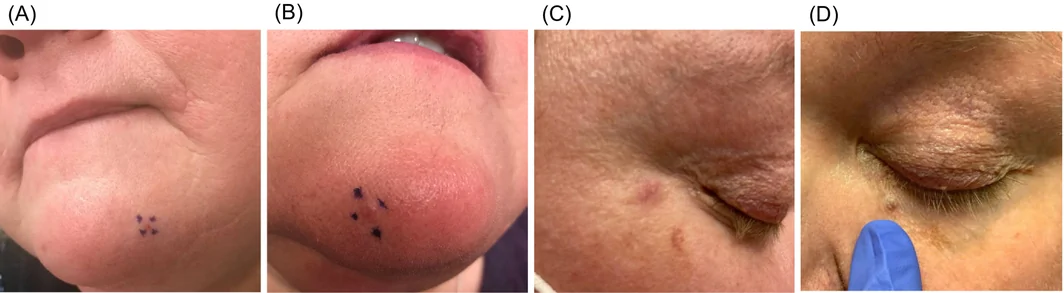
Skin to red‐colored papules asynchronously presented on the bilateral chin (A and B), the right lateral canthus (C), the right cheek (photo not shown), and the left lower eyelid (D).

**FIGURE 2:**
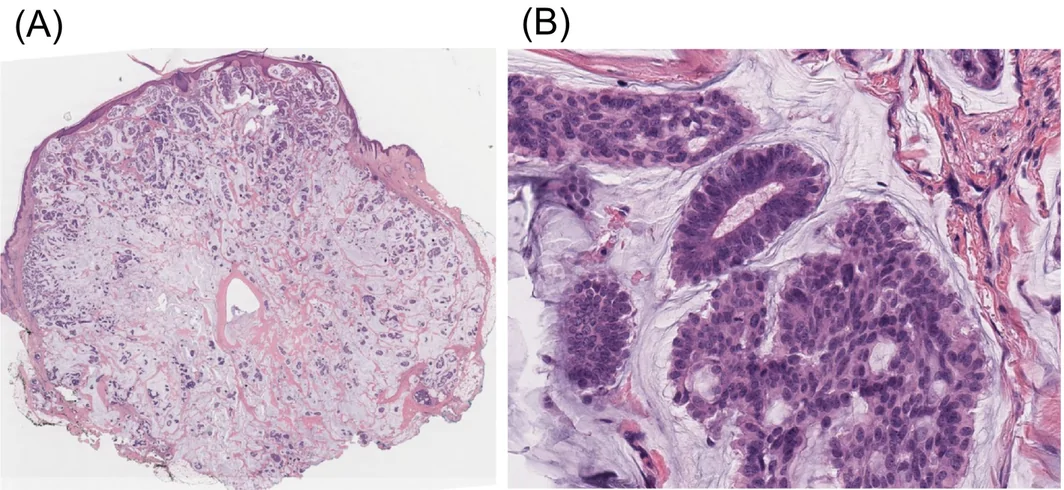
MCS of the nodule on the left chin. (A) Well‐circumscribed dermal‐based nodule with lobular architecture. Tumor nests are floating in pools of mucinous ground substance (hematoxylin–eosin stain; original magnification: ×20). (B) Tumor cells contain plump ovoid nuclei with a crowded chromatin pattern and occasional mitotic figures (hematoxylin–eosin stain; original magnification: ×200).

A 55‐year‐old woman was diagnosed with MCS on her left chin 2 years prior to initial presentation in our clinic. The lesion was treated by Mohs micrographic surgery (MMS), resulting in clear margins (Figures 1A and 2A,B). She was previously healthy aside from obesity with obstructive sleep apnea and had no personal or family history of developmental delay.

**FIGURE 3:**
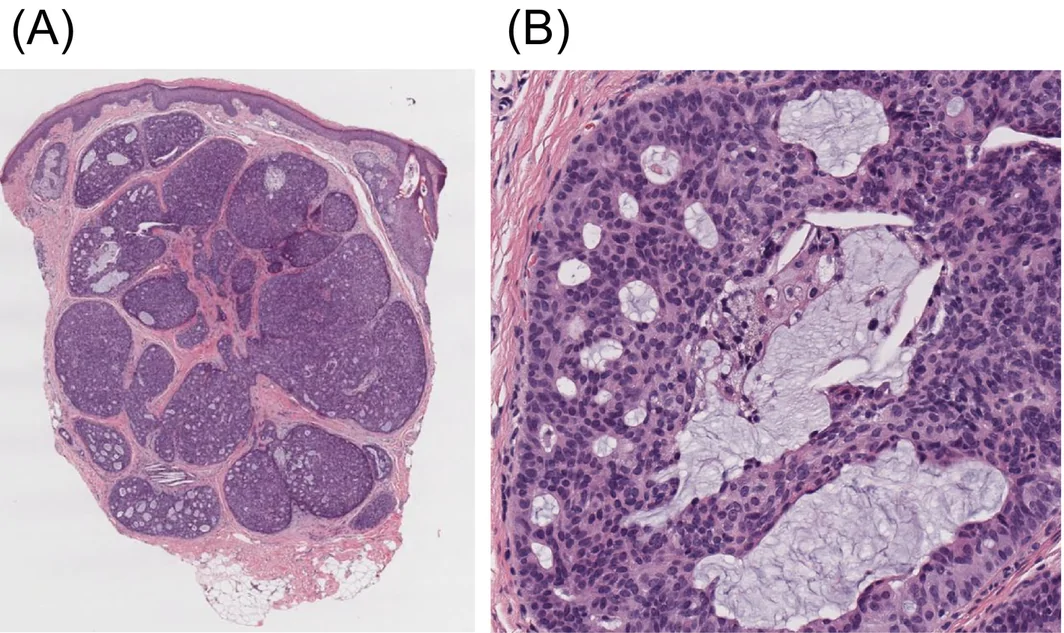
The nodule on the right chin exhibits features of EMPSGC. (A) A well‐demarcated dermal‐based nodular lesion is composed of multiple lobules of basaloid cells (hematoxylin–eosin stain; original magnification: ×20). (B) Tumor cells display enlarged nuclei and nuclear pleomorphism. There is mucin within the tumor islands (hematoxylin–eosin stain; original magnification: ×140).

**FIGURE 4:**
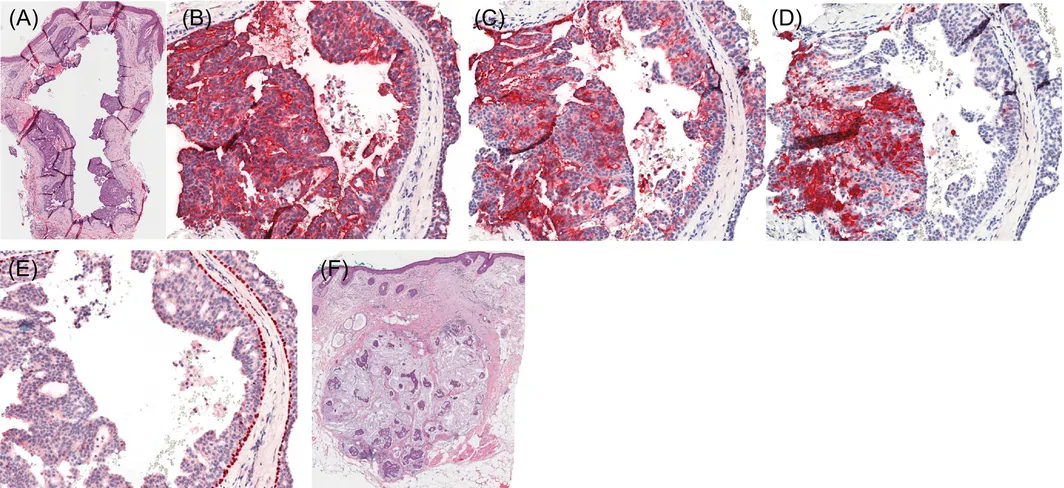
Initial biopsy of the right lateral canthus lesion shows EMPSGC (A–E), and Mohs at the same site reveals MCS (F). (A) A biopsy specimen shows a neoplasm composed of multiple basaloid tumor islands (hematoxylin–eosin stain; original magnification: ×15). (B) EMA stains are diffusely positive in tumor cells (original magnification: ×125). CEA (C) and chromogranin (D) stains demonstrate areas of positivity (original magnification: ×125). (E) P63 stains highlight the cells at the periphery of the tumor islands (original magnification: ×125). (F) The MMS debulk specimen shows histopathologic features that are more typical of MCS (hematoxylin–eosin stain; original magnification: ×10).

A new reddish shiny papule appeared on her right chin (Figure 1B). A biopsy showed basaloid solid and cystic nodules occupying the dermis, with extracellular mucin in the tumor lobules, consistent with EMPSGC (Figure 3A,B). A systemic workup including PET/CT scan showed no metastases nor other primary malignant lesions, although pancreatic intraductal papillary mucosal neoplasms were detected. Four years later, another biopsy from the right lateral canthus (Figure 1C) showed EMPSGC (Figure 4A–E); the subsequent MMS procedure at this site revealed features more typical of MCS (Figure 4F).

**FIGURE 5:**
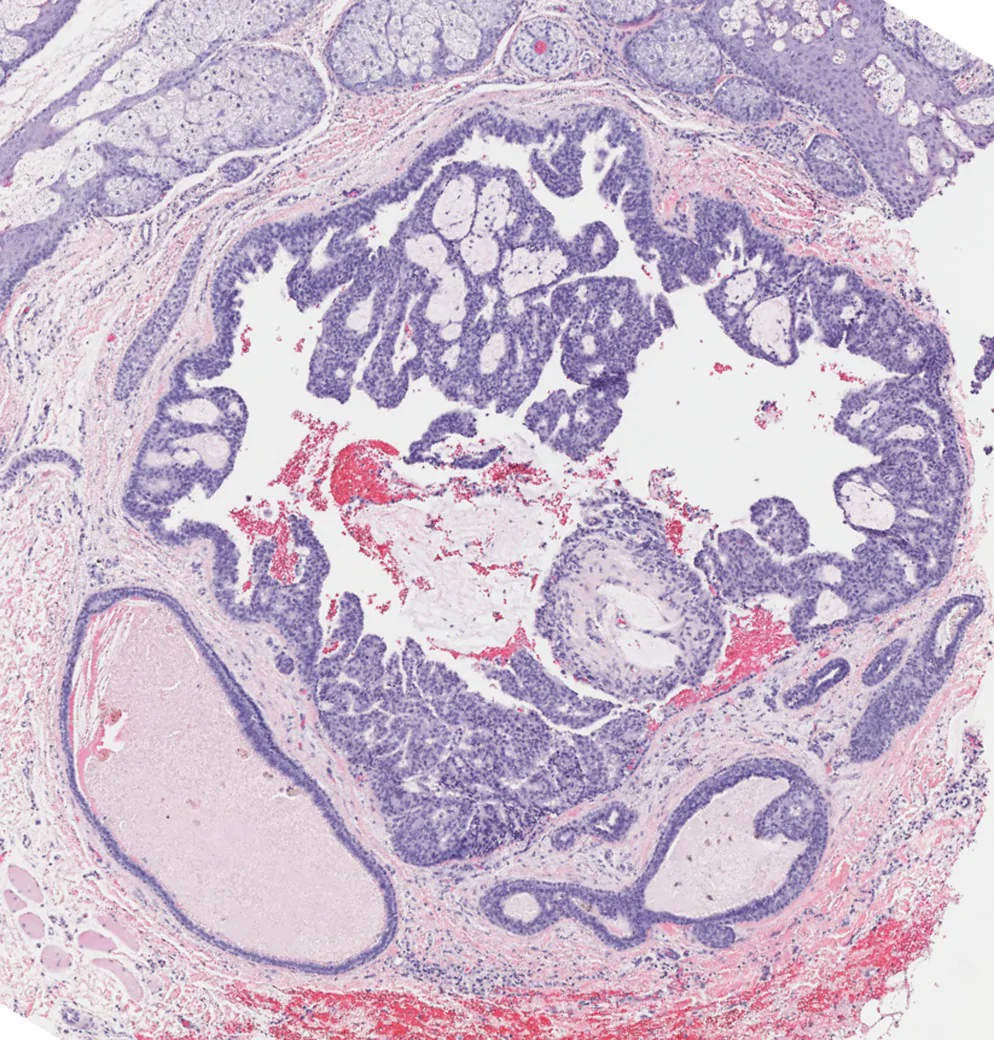
The nodule on the right cheek was diagnosed as EMPSGC (hematoxylin–eosin stain; original magnification: ×40). The tumor cells are negative for chromogranin and synaptophysin (data not shown).

**FIGURE 6:**
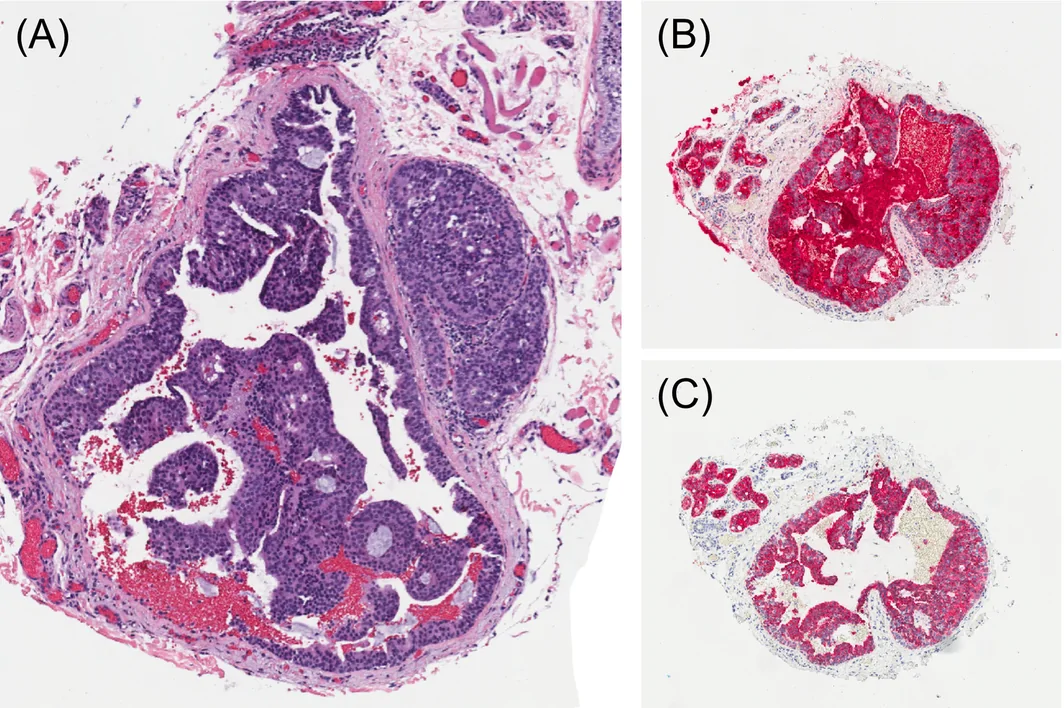
(A) The left lower eyelid lesion was diagnosed as EMPSGC (hematoxylin–eosin stain; original magnification: ×50). GCDFP15 (B) and CK7 (C) stains are positive in tumor cells (original magnification: ×30). Chromogranin and synaptophysin are negative (data not shown).

In the next 3 years, new histopathologically confirmed EMPSGC lesions sequentially occurred on the right cheek and the left lower eyelid. Figure 1D shows clinical presentation on the left lower eyelid. Figures 5 and 6A–C show the histopathologic findings of the right cheek and left lower eyelid lesions, respectively. Both sites were treated with MMS. The tumors were positive for EMA and CEA stains (Figure 4B,C). Chromogranin staining was positive in a subset of cells in the right lateral canthus lesion (Figure 4D) but was negative in other lesions (not shown). P63 stains highlighted the peripheral cells of the tumor islands (Figure 4E). GCDFP15 and cytokeratin 7 stains were positive (Figure 6B,C). Next generation sequencing (PennSeq Solid Cancer Sequencing Panel, 183 genes) on the right canthus lesion and the right cheek lesion revealed the same missense variant in the *ATRX* gene (NM_000489.6: c.2924A>G, p.D975G, variant allele fraction (VAF): 47% and 49%, respectively). The NGS assay did not detect any other abnormalities.

Following the last MMS, the patient is still under active surveillance. To date, no metastasis or local recurrence has been found in the 12 months since the last MMS.

## 3 Discussion

Histopathologically, EMPSGC exhibits an expansile growth pattern, which is thought to correspond to an intermediate stage in the multistep progression from benign sweat duct cysts to carcinoma in situ and invasive mucinous carcinoma [9, 10, 11]. Au et al. reported that 35.7% of EMPSGC cases are associated with invasive neuroendocrine MCS components through a review of the literature, as seen in the present case [11]. As with SPC exhibiting expression of neuroendocrine and breast‐type markers, EMPSGC is commonly positive for focal and variable synaptophysin and chromogranin and more diffuse INSM1 staining. There is also frequent CAM 5.2, EMA, pCEA, CK7, estrogen receptors, progesterone receptors, GCDFP‐15, and GATA immunoreactivity [9, 12]. The histopathological finding of an in situ component surrounded by myoepithelial cells staining with SMA, calponin, p63/p40, and S100 stains is helpful to distinguish EMPSGC from metastasis from another organ system, including the breast and gastrointestinal tract. However, because of its indolent behavior, it is now accepted that an expansile growth pattern should still be considered an in situ lesion even if the myoepithelial rim is lost, and extracellular pools of mucin and/or infiltration of tumor glands indicate progression to invasive MCS [13]. A systemic workup should be conducted to rule out metastasis from other organs.

Our patient has been diagnosed with five separate lesions of EMPSGC and MCS distributed beyond the periorbital region on the face. To date, six manuscripts have reported eight cases of distinct multicentric MCSs, EMPSGCs, or EMPSGCs with MCSs on the eyelid or the cheek (Table 1) [2, 3, 4, 5, 6, 7]. In addition to these published cases, there are reports of patients with EMPSGC with synchronous noncutaneous tumors, including an invasive mucinous ductal carcinoma of the breast and an intraductal papilloma of the breast [7, 14]. Most previous reports describe localized bilateral periorbital lesions, suggesting a possible “field effect” (i.e., a field of cellular and molecular alteration predisposing to the development of neoplasms within that field) as a potential cause of multicentricity. However, the more widespread distribution observed in the present case and the co‐occurrence of analogous tumors in other organs in other reports might indicate an underlying susceptibility to developing these tumors, such as a genetic predisposition including germline variants, environmental exposures, and lifestyle factors, which facilitate progression through common carcinogenic pathways [15].

**TABLE 1:** Summary of multicentric MCSs, EMPSGCs, or EMPSGCs with MCSs.

First author	Age	Sex	Site and diagnosis	Diagnostic timing of each lesion	Associated noncutaneous tumors
Furst [2]	70	F	Rt. lower eyelid, MCS Rt. Cheek, MCS	Initial visit^a^	None
Bertagnoli [3]	55	F	Lt. lateral lower eyelid, MCS Rt. lateral lower eyelid, MCS	NA	None
Burris [4]	71	F	Lt. upper eyelid, MCS. Rt. upper eyelid, MCS	One year after the initial diagnosis, a second lesion appeared.	None
Stiegler [5]	68	F	Lt. upper eyelid, MCS Lt. central lower eyelid, MCS Lt. medial lower eyelid, MCS	Initial visit^a^	None
Nishimoto [6]	71	F	Lt. upper eyelid, MCS Lt. cheek, EMPSGC Rt. cheek, EMPSGC	Initial visit^a^	Invasive ductal carcinoma of the breast. Uterine corpus cancer
Ravi [7]	59	F	Lt. cheek, EMPSGC and MCS Lt. upper eyelid, EMPSGC and MCS Rt. lower eyelid, MCS	Initial visit^a^	None
	79	M	Rt. lower medial eyelid, EMPSGC Rt. lower lateral eyelid, EMPSGC and MCS	Initial visit^a^	None
	75	F	Lt. lower eyelid, EMPSGC Lt. upper eyelid, EMPSGC	Three years after the initial diagnosis, a second lesion appeared.	Mucinous carcinoma, Rt. breast. Endometrioid endometrial adenocarcinoma
Our case	55	F	Lt. chin, MCS Rt. chin, EMPSGC Rt. lateral canthus, EMPSGC and MCS Rt. cheek, EMPSGC Lt. lower eyelid, EMPSGC	Over the course of 8 years, each lesion developed.	Pancreatic intraductal papillary mucosal neoplasms

Abbreviations: EMPSGC, endocrine mucin‐producing sweat gland carcinoma; F, female; Lt., left; MCS, mucinous carcinoma of the skin; M, male; Rt., right.

^a^ All lesions were present at the initial visit.

In the present case, we attempted targeted NGS on formalin‐fixed paraffin embedded tissue from all of the specimens received in our laboratory (one had previously been sent to an outside laboratory). Only two specimens yielded sufficient material for analysis; both revealed the same missense ATRX (NM_000489.6) variant. The *ATRX* gene, located on the X chromosome, encodes the alpha thalassemia/mental retardation syndrome X‐linked protein (ATRX), which is a helicase involved in chromatin remodeling [16, 17]. Loss‐of‐function variants are associated with genomic instability and oncogenesis, and are strongly associated with the alternate lengthening of telomeres (ALT) tumor phenotype [18]. Inactivated somatic mutations or pathogenic variants (PVs) of *ATRX* are identified in many cancers including gliomas, neuroblastomas, and pancreatic neuroendocrine tumors (PanNETs) [18, 19, 20]. A recent immunohistochemical analysis reported that the loss of ATRX or its interacting protein, death domain‐associated protein (DAXX), expression was significantly associated with lymphatic invasion in SPC [21].

Alpha Thalassemia X‐linked (ATRX) syndrome is caused by a variety of disruptive germline mutations in the *ATRX* gene. While it is characterized by severe developmental delay, facial dysmorphism, hypotonia, and abnormalities in the skeletal, genitourinary, and hematopoietic systems in male individuals, most female carriers of the *ATRX* gene mutation exhibit a skewing X chromosome offset inactivation with preferential inactivation of the X chromosome carrying the ATRX mutation and do not have developmental delay [22]. The variant identified in the present case has not been reported in ATRX syndrome or cancers and is classified as a variant of unknown clinical significance according to Human Gene Variation Significance (HGVS). The VAF at close to 50% (47%–49%) in multiple tumors may suggest a germline origin of this variant in the current patient. While germline testing for *ATRX* gene mutation was considered in our patient, this was not pursued after discussion with medical genetics at our institution because of the unknown significance of the *ATRX* somatic variants and the lack of data supporting cancer predisposition in female heterozygous *ATRX* carriers. The role of *ATRX*, if any, in EMPSGC/MCS carcinogenesis remains to be elucidated. Further genetic and molecular studies in other patients with multiple EMPSGC may be helpful in evaluating for this possibility.

## Ethics Statement

The authors have nothing to report.

## Conflicts of Interest

The authors declare no conflicts of interest.

## Data Availability

The data that support the findings of this study are available on request from the corresponding author. The data are not publicly available due to privacy or ethical restrictions.
